# Obesity-Mediated Inflammation and Its Influence on Inflammatory Bowel Disease: Pathophysiology, Clinical Impact, and Therapeutic Implications

**DOI:** 10.3390/biom15081185

**Published:** 2025-08-18

**Authors:** Diego Casas-Deza, Santiago García-López, Vanesa Bernal-Monterde, Cristina Polo-Cuadro, Carmen Yagüe-Caballero, José M. Arbones-Mainar

**Affiliations:** 1 Gastroenterology Department, Miguel Servet University Hospital, 50009 Zaragoza, Spain; diegocasas8@gmail.com (D.C.-D.); sgarcia.lopez@gmail.com (S.G.-L.); criscpc05@gmail.com (C.P.-C.); carmenyaguecaballero@gmail.com (C.Y.-C.); 2 Health Investigation Institute Aragón (IIS-A), 50009 Zaragoza, Spain; 3 Adipocyte and Fat Biology Laboratory (AdipoFat), Translational Research Unit, Miguel Servet University Hospital, 50009 Zaragoza, Spain; 4 Instituto Aragones de Ciencias de la Salud (IACS), 50009 Zaragoza, Spain; 5 CIBER Fisiopatología Obesidad y Nutrición (CIBERObn), Instituto Salud Carlos III, 28029 Madrid, Spain

**Keywords:** adipose tissue, Crohn’s disease, ulcerative colitis, inflammation, visceral adiposity, dysbiosis

## Abstract

Obesity and inflammatory bowel disease (IBD) are two chronic conditions whose prevalence continues to rise globally. Emerging evidence suggests a bidirectional interplay between them, mediated by shared pathophysiological pathways. This narrative review explores the mechanisms Ilinking obesity to IBD development and progression, focusing on the role of adipose tissue dysfunction. Both diseases exhibit intestinal dysbiosis, low-grade systemic inflammation, and impaired epithelial barrier integrity, contributing to immune activation. Visceral adiposity, particularly mesenteric fat, acts as an immunometabolic organ producing cytokines and adipokines that may exacerbate intestinal inflammation. In Crohn’s disease, mesenteric fat expansion, or “creeping fat”, is associated with transmural inflammation, fibrosis, and luminal narrowing. Epidemiological data on obesity as a risk factor for IBD remain inconsistent due to methodological heterogeneity and confounders. Similarly, the impact of obesity on IBD outcomes, including disease activity, phenotype, and the need for surgery, is debated. While mesenteric surgical approaches like Kono-S anastomosis showed initial promise in reducing recurrence, recent randomized trials offer conflicting results. Finally, metabolic drugs such as statins, metformin, and GLP-1 receptor agonists have demonstrated anti-inflammatory properties with potential utility in IBD management. Prospective studies are warranted to elucidate the clinical significance of obesity and metabolic dysfunction in IBD and evaluate targeted therapeutic strategies.

## 1. Introduction

Inflammatory bowel disease (IBD) is a chronic condition comprising two main entities: ulcerative colitis (UC) [1] and Crohn’s disease (CD) [2]. These are immune-mediated pathologies consisting of inflammatory processes primarily affecting the digestive tract, alternating between remission periods and inflammatory activity. There are some important differences between these two entities. UC is characterized by continuous involvement of the colonic mucosa, to a variable extent, starting at the anus. CD, on the other hand, is a discontinuous, transmural inflammation that can discontinuously affect any part of the digestive tract, from the mouth to the anus. The incidence and prevalence of IBD are increasing, particularly in industrialized countries, where its prevalence is approaching 1%. While regions with a traditional lifestyle still show lower rates, emerging data suggest that developing regions undergoing westernization may also experience a rising trend (3). This increase stems from sociodemographic changes associated with economic development, including, among others, ultra-processed-food-rich diets, improved hygiene conditions, smoking, and a sedentary lifestyle [3,4,5].

The causes of IBD are only partly known. The current model involves a combination of genetic predisposition, with more than 200 variants identified, and environmental exposures. These include early-life factors such as breastfeeding and antibiotic use, as well as later influences like diet, medications, smoking, and the composition of the gut microbiota [1,2]. In recent years, obesity and adipose tissue have emerged as relevant risk factors for IBD [6,7,8]. This relationship is more relevant in CD, where due to the transmural involvement that takes place, the interaction with the mesenteric adipose tissue is much closer. Obesity rates continue to rise in developed countries and are linked to other digestive disorders, including metabolic liver disease, acute pancreatitis, and gastrointestinal cancers [9]. Visceral fat in particular is metabolically active and has endocrine and immune functions that promote inflammation [10]. It also interacts with key IBD-related factors such as diet, sedentary behavior, and the gut microbiome [11,12].

Obesity often coexists with metabolic conditions like diabetes, hypertension, and dyslipidemia. Together, these form metabolic syndrome, which has also been associated with IBD, with an estimated 20% of IBD patients having a simultaneous diagnosis compatible with metabolic syndrome [13,14,15].

The relevance of this link is heightened by the sharp rise in childhood obesity, 1.5 times higher from 2012 to 2023 compared to that from 2000 to 2011, especially in high-income countries [16,17]. In addition, IBD is an independent risk factor for the development of another disease closely linked to metabolic syndrome: Metabolic-Dysfunction-Associated Steatotic Liver Disease [18].

Despite growing interest, there are still several aspects that remain unclear. Some findings, such as the possible implication of obesity as a risk factor for the development of IBD [19] and the usefulness of mesentery surgery, have conflicting results [20]. Others, such as the possible application to IBD of the treatments used for metabolic pathologies, require further investigation despite promising data. Assessing the relationship between obesity and IBD is particularly difficult because of the many confounding factors linked to both conditions, and assessing what is a cause and what is a consequence is particularly complex [21]. In this narrative review, we examine the different mechanisms linking obesity to IBD, with a special focus on the adipose tissue and its dysfunction.

## 2. Shared Pathophysiological Mechanisms Between Obesity and IBD

The hypothesis that obesity contributes to IBD requires support from pathophysiological mechanisms. A parallel rise in their incidence is not enough since both conditions share common risk factors such as diet and physical inactivity, which may explain the association by coincidence. Below, we review mechanisms common to both diseases that may support a causal link. IBD involves several proposed pathways beyond the classic model of insulin resistance. Determining whether obesity is a cause or a consequence remains challenging.

### 2.1. Intestinal Dysbiosis

Both obesity and IBD are associated with quantitative and qualitative alterations in the intestinal microbiota, known as dysbiosis [22]. Both conditions consistently show increased *Proteobacteria phylum* bacteria (including pathogens like *E. coli*) and species like *Ruminococcus gnavus*, along with decreased beneficial bacteria like Clostridiales (the leptum subgroup) and *Faecalibacterium prausnitzii* [22,23,24]. Obesity also features increases in *Firmicutes* and reductions in *Actinobacteria*, while IBD typically shows global Firmicutes loss and Bacteroidetes changes, with pathogenic *Enterobacteriaceae* proliferation [23,24,25].

These microbial changes can compromise intestinal barrier function: reduced commensal bacteria producing short-chain fatty acids (SCFAs, like butyrate) decrease the energy sources for the colonocytes [26,27] and affect intestinal epithelial integrity [28,29]. A lower butyrate supply, crucial to mucosal health, is associated with increased intestinal permeability, while certain SCFAs (e.g., acetate) help preserve barrier function and prevent bacterial toxin translocation. Additionally, dysbiosis linked to obesity and IBD promotes the accumulation of lipopolysaccharides (LPSs) from Gram-negative bacteria, which can cross an altered intestinal barrier and activate the innate immune system, contributing to chronic low-grade inflammatory states in both obesity and IBD [30].

### 2.2. Chronic Immune Activation and Pro-Inflammatory Cytokines

Obesity is now recognized as a state of systemic inflammation [31,32]. The visceral fat functions as an immune–endocrine organ, infiltrated by activated macrophages, mainly of the M1 type [33], and other immune cells. It produces pro-inflammatory cytokines such as TNF-α, IL-1β, and IL-6, along with adipokines that sustain chronic inflammation. The levels of many of these cytokines may be elevated at the systemic level [34]. Obesity has also recently been reported to result in mitochondrial dysfunction, which promotes the secretion of pro-inflammatory cytokines by the dendritic cells. This phenomenon results in an increase in Th17 cells, which are crucial in intestinal inflammation phenomena [35]. Another interesting mechanism involves calcium (Ca^2+^) signaling. Dysregulated Ca^2+^-mediated pathways have been implicated in both adipogenesis and inflammation, particularly in the context of obesity-associated insulin resistance. These molecular interactions highlight a potential link between calcium homeostasis and the development of metabolic dysfunction in the adipose tissue [36,37,38]. In IBD, both innate and adaptive immune responses are abnormally activated in the intestinal mucosa, leading to the release of a similar set of cytokines, including TNF-α, IL-6, IL-12/23, and interferon-γ, which drive tissue injury [39].

These overlapping inflammatory pathways point to a shared mechanism. The interaction between an inflamed gut wall and the visceral fat may not only sustain but also amplify inflammation, creating a feedback loop. However, it remains unclear whether adipose inflammation drives intestinal disease or results from it.

### 2.3. The Role of Adipokines

Adipose-tissue-derived hormones also modulate the immune response in obesity and IBD. For example, leptin, with increased expression in the mesenteric fat in IBD patients [40], is pro-inflammatory, enhancing macrophage activation, along with TNF-α, and promoting Th1 and Th17 lymphocyte responses and the macrophages [41]. High local leptin levels can activate the NF-κB pathway in intestinal epithelial cells, promoting neutrophil infiltration and colonic inflammation [42,43].

In contrast, adiponectin typically has anti-inflammatory effects by stimulating the release of regulatory cytokines (IL-10, IL-1Ra) from the macrophages and lymphocytes [42,44]. Other adipokines like resistin and visfatin, which are elevated in obesity, act as pro-inflammatory factors and have been found to be increased in patients with active IBD [43,45]. In the case of resistin, this role could be due to its function as an inducer of the Toll-like receptor-4/NF-κB [46]. In summary, systemic subclinical inflammation mediated by adipokines converges with IBD inflammatory pathways, creating a shared immunological storm that could exacerbate intestinal pathology.

### 2.4. Intestinal Barrier Integrity

The disruption of intestinal barrier integrity may represent a key mechanistic link between obesity and IBD [47]. In obesity, low-grade metabolic inflammation is linked to increased permeability and systemic exposure to bacterial products like LPS, which further activate the immune system. In IBD, chronic inflammation disrupts the epithelial tight junctions and facilitates microbial translocation, reinforcing the inflammatory cycle [48]. This phenomenon is partly driven by a shared loss of protective, butyrate-producing microbiota, which compromises the epithelial energy supply and tight junction maintenance [24,49]. Ensuing barrier impairment facilitates the translocation of bacterial components, such as lipopolysaccharides, into the lamina propria and the systemic circulation, promoting inflammation locally and systemically. In obesity, this contributes to chronic low-grade inflammation, while in IBD, it perpetuates a cycle of mucosal injury and immune dysregulation. Thus, altered barrier function emerges as a potential pathophysiological intersection between these seemingly distinct diseases [50].

**Figure 1:**
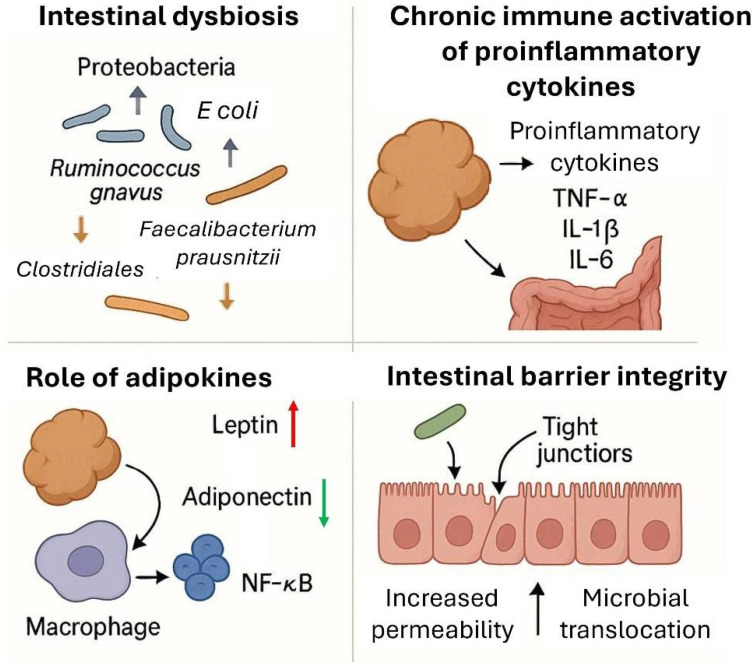
Obesity-associated inflammation contributes to the pathophysiology of inflammatory bowel disease (IBD) through multiple interrelated mechanisms. Intestinal dysbiosis in obesity is characterized by an increased abundance of Proteobacteria and pro-inflammatory taxa such as *Ruminococcus gnavus* and *E. coli*, along with a reduction in anti-inflammatory bacteria like *Faecalibacterium prausnitzii* and *Clostridiales* (**top left**). This dysbiotic state promotes chronic immune activation and the release of pro-inflammatory cytokines including tumor necrosis factor alpha (TNF-α), interleukin-1b (IL-1b), and interleukin-6 (IL-6) (**top right**). Adipokines, such as increased leptin and decreased adiponectin, further exacerbate inflammation by activating the macrophages and promoting NF-κB signaling (**bottom left**). These inflammatory processes impair intestinal barrier integrity, weakening the tight junctions and increasing epithelial permeability, thereby facilitating microbial translocation and sustaining gut inflammation (**bottom right**). This figure was created in part with DALL·E 3 (OpenAI).

Together, obesity and IBD involve similar alterations in gut barrier function and immune stimulation, supporting shared inflammatory pathophysiology. The processes mentioned above are represented in Figure 1

## 3. Obesity as a Risk Factor for IBD Development

Recent epidemiological studies place the prevalence of obesity and overweight in IBD patients at around 40–50% [51,52], consistent with the increase in the prevalence of this condition in the global population. The classic presentation of IBD, particularly CD, with weight loss, a low body mass, and even cachexia, is now uncommon in adults and largely restricted to isolated cases.

Recent data indicate that obesity might influence the risk of developing IBD, especially CD. However, this evidence remains inconsistent (Table 1). Several studies suggest that obesity, assessed through body mass index (BMI), may increase the risk of developing IBD. For example, Khalili et al. [53] and Mendall et al. [54], both conducting studies in cohorts of young or reproductive-age women, reported an association between a higher BMI and increased IBD risk. In the former, this association was observed for both CD and UC, while in the latter, it was limited to CD. Similarly, Jensen et al. [55], in a pediatric cohort, found a positive correlation between a higher BMI and CD risk but not the same relationship for UC. In contrast, studies conducted in general-population cohorts, such as those by Chan et al. [56] and Harpsøe et al. [57], did not find significant differences in IBD incidence across BMI categories. More recently, a population-based study in Spain by Cañete et al. [58] identified severe obesity and a history of bariatric surgery as independent risk factors for IBD.

Several factors may account for the heterogeneity in these findings. First, the method used to assess obesity is a major limitation. BMI is a crude metric that does not distinguish between subcutaneous and visceral fat, with the latter being more metabolically active. It may also misclassify individuals with a high muscle mass as obese and fail to capture inter-individual variability in adipose tissue function, which can be significant, even among patients with similar BMI values. Recent recommendations propose using BMI solely as a population-level proxy, adjusted for sex, age, and ethnicity. For a clinical diagnosis, a more comprehensive assessment of body composition and metabolic function is required [59]. Second, most studies rely on population-based databases, which are prone to various biases. Third, the characteristics of the study populations differ. Studies reporting obesity as a risk factor often focus on selected groups, particularly younger individuals and women, whereas studies with negative findings include broader, more heterogeneous populations. It is possible that obesity during early life exerts a stronger influence that diminishes with age. Additionally, the available data suggest that the risk of IBD increases with the severity of obesity, as highlighted in the study by Cañete et al. [58].

## 4. The Impact of Obesity on the Clinical Course and Outcomes of IBD

In addition to its possible role in disease onset, obesity has also been proposed as a risk factor for the development of more complex or aggressive IBD phenotypes, as well as increased disease activity and flare frequency. However, the available evidence remains inconsistent and inconclusive.

Regarding phenotype complexity, Pringle et al. reported that individuals with obesity were less likely to develop a penetrating phenotype in CD, while the risk of stricturing disease or perianal involvement was similar between groups [60]. Another study involving 297 patients with CD found no significant differences in the prevalence of penetrating or stricturing phenotypes between individuals with and without obesity [61]. Some of these studies have also suggested a higher incidence of colonic involvement in individuals with obesity, although this finding has not been consistently replicated. In UC, the results are similarly mixed. While some studies have linked obesity with increased disease activity, others, such as that by Stabroth-Akil et al. [62], have observed a lower prevalence of pancolitis in individuals with obesity compared to that in those with a BMI in the normal range.

These inconsistencies likely reflect the same methodological limitations seen in the studies assessing obesity as a risk factor for IBD onset. Most available studies have relied solely on BMI to define obesity, despite its inability to distinguish fat distribution or assess adipose tissue function. Furthermore, key confounding variables closely associated with obesity, such as lifestyle and dietary habits, are often overlooked. In this context, recent evidence may offer additional insights. A prospective cohort study by García-Mateo et al. [63] showed that adherence to a healthy lifestyle, including regular physical activity and a Mediterranean diet, was associated with a lower risk of disease flares and reduced corticosteroid use. These findings reinforce earlier studies that independently linked physical activity and diet to improved outcomes in individuals with IBD [64,65,66]. Additional research suggests that obesity may negatively impact other aspects of IBD, including reduced quality of life (Jain et al.), prolonged hospital stays [67], increased rates of perianal complications [68], a higher incidence of colorectal cancer [69], and an earlier need for surgical intervention [70].

In summary, while the current evidence is not definitive and some studies report more favorable outcomes in individuals with obesity, the overall data suggest a potential association between obesity and a worse disease progression in IBD. This effect may be partly mediated by modifiable factors commonly linked to obesity, such as physical inactivity or unhealthy dietary patterns, rather than obesity per se.

## 5. Visceral Adipose Tissue, Creeping Fat, and IBD

**Figure 2:**
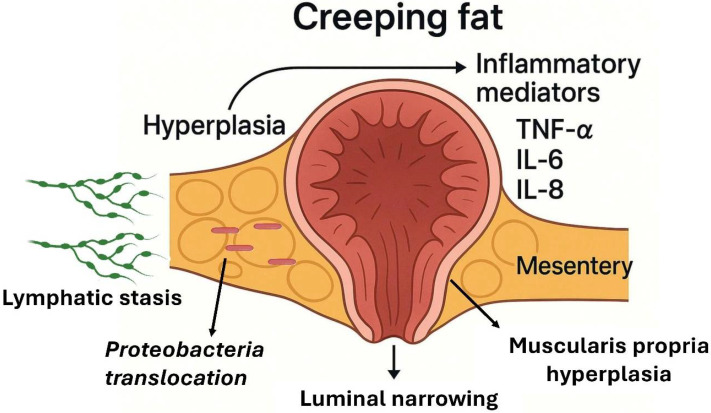
Creeping fat and its pathophysiological role in intestinal inflammation and fibrosis. Creeping fat is characterized by mesenteric adipose tissue hyperplasia surrounding inflamed intestinal segments in Crohn’s disease. Lymphatic stasis facilitates the translocation of Proteobacteria into the adipose tissue, triggering an immune response and the secretion of pro-inflammatory cytokines, including tumor necrosis factor-alpha (TNF-α), interleukin-6 (IL-6), and interleukin-8 (IL-8). These mediators promote further adipose tissue remodeling and inflammation. In parallel, muscularis propria hyperplasia contributes to luminal narrowing, exacerbating intestinal obstruction. The above figure was created in part using DALL·E 3 (OpenAI).

A distinct manifestation of the interaction between the adipose tissue and IBD is the expansion of mesenteric fat, known as “creeping fat” [71]. This feature is characteristic of CD and involves hypertrophy of the mesenteric adipose tissue that encases inflamed intestinal segments, progressing from the mesenteric border along the serosal surface [72]. First described by B.B. Crohn in 1932, this phenomenon, also referred to as “fat wrapping”, is considered almost pathognomonic of the transmural form of CD (Figure 2).

Histologically, hypertrophied mesenteric fat in CD is considered a complex immunological zone and consists of adipocytes, preadipocytes, fibroblasts, mesenchymal stem cells, endothelial cells, and abundant infiltrated immune cells (macrophages, lymphocytes) [73,74,75]. Creeping fat is characterized by an increased presence of M2 macrophages compared to M1 macrophages. This M2-polarized state is thought to promote intestinal fibrosis by releasing high levels of pro-fibrotic cytokines such as transforming growth factor-beta (TGF-β). In contrast, the M1 macrophages are more prevalent in the lamina propria and the inflamed ileal mucosa. In patients with ileal CD, the total percentage of T-regulatory (Treg), Th1, and Th17 cells is significantly higher in the creeping fat than that in the adjacent mucosa. Creeping fat also contains a greater proportion of T cells compared to that in non-CD mesenteric adipose tissue [75,76]. Functional analyses of the differentially expressed genes in creeping fat show enrichment of immune-related pathways and the downregulation of genes involved in lipid metabolism, including adipogenesis. This activated adipose tissue secretes a broad range of pro-inflammatory mediators, including cytokines (e.g., TNF-α, IL-6, IL-8), chemokines, adipokines, and growth factors, which may diffuse into the neighboring intestinal wall. This is consistent with the systemic elevation of several inflammatory markers, such as macrophage migration inhibitory factor (MIF), IL-16, interferon-gamma (IFN-γ), IL-1β, and TNF-α, observed in patients with CD compared to those in healthy controls [77,78]. Additionally, by interposing between the serosa and the muscularis propria of the intestine, the creeping fat mass makes direct contact with the muscle layers, associated with muscular hyperplasia and wall fibrosis [79,80]. This triad of enveloping mesenteric fat, muscle thickening, and transmural inflammation contributes to intestinal stenosis formation in CD. Surgical specimen studies confirm that fat wrapping is exclusive to CD (not observed in resections for other pathologies like cancer or ischemia) and positively correlated with intense transmural inflammation, fibrosis, and luminal narrowing in the affected segment. It has been postulated that creeping fat in CD arises more from hyperplasia (an increase in number) of the adipocytes than from their hypertrophy, given that up to four times more mesenteric adipocytes have been found in diseased Crohn’s segments compared to those in normal mesentery [71]. The release of free fatty acids by this creeping fat in turn induces hyperplasia of the muscle cells of the intestinal muscularis propria layer. This finding is one of the most prominent histological changes in fibrosing stenosis in CD [81].

Interestingly, the distribution of creeping fat varies according to disease location: it is prominent in ileal CD (ileum involvement) and much less in colonic CD or UC [82]. In ileal samples, creeping fat contains more fibrotic tissue and T lymphocytes than those in mesenteric fat obtained from the colon of CD or UC patients, suggesting differences in the adipose response according to intestinal context [77]. This supports the notion that ileal and colonic CD may be biologically distinct entities in their interactions with the adipose tissue. Creeping fat formation mechanisms are linked to chronic intestinal inflammation and altered mesenteric lymphatic circulation. Transmural inflammation in CD could lead to insufficient or blocked lymphatic drainage; poor intestinal lymphatic drainage has been described to favor local mesenteric adiposity accumulation. Indeed, dilated or obstructed mesenteric lymphatics have been observed in CD patients during surgery [83]. One proposed model suggests that lymphatic stasis and inflammation drive adipocyte proliferation. Under inflammatory conditions, activated smooth intestinal muscle cells secrete extracellular matrix components such as fibronectin. These molecules act through integrin signaling to recruit and promote the migration of preadipocytes from the surrounding mesentery, contributing to the expansion of creeping fat around inflamed intestinal segments [84]. Supporting this, a recent study demonstrated that the muscularis propria cells in CD can induce creeping fat formation via these pro-adipogenic signals [81].

The mesenteric adipose tissue in CD may not be sterile. Several studies have identified a distinct microbial signature within the creeping fat and mesenteric fat, primarily composed of Proteobacteria, while the subcutaneous fat shows no such colonization [76,85]. The abundance of Proteobacteria in the mesenteric fat correlates with markers of active inflammation, including elevated fecal calprotectin and serum C-reactive protein levels [86]. Moreover, individuals with CD in remission have lower levels of pathogenic bacteria in the mesenteric fat compared to these levels in those with active disease.

All of the above suggests that bacterial translocation through a compromised intestinal barrier may seed the mesenteric fat, creating a persistent antigenic stimulus that sustains local inflammation [76,87]. Paradoxically, some authors propose that this inflammatory remodeling of the adipose tissue may serve a defensive role: creeping fat could act as an immunological barrier, isolating inflamed intestinal segments and limiting the spread of bacteria beyond the gut [71]. However, this same response can have deleterious consequences, including fibrosis and luminal narrowing, which may contribute to the development of a stricturing phenotype [80].

## 6. Mesenteric Surgery: Unfulfilled Promises

Based on the previously described pathophysiological mechanisms, a hypothesis has emerged: surgical intervention in the mesentery in CD, particularly in ileal resections, might improve long-term outcomes and reduce postoperative recurrence [20]. Two main strategies have been proposed: mesenteric exclusion through specific anastomotic techniques, notably Kono-S anastomosis, and extended mesenteric resection.

**Figure 3:**
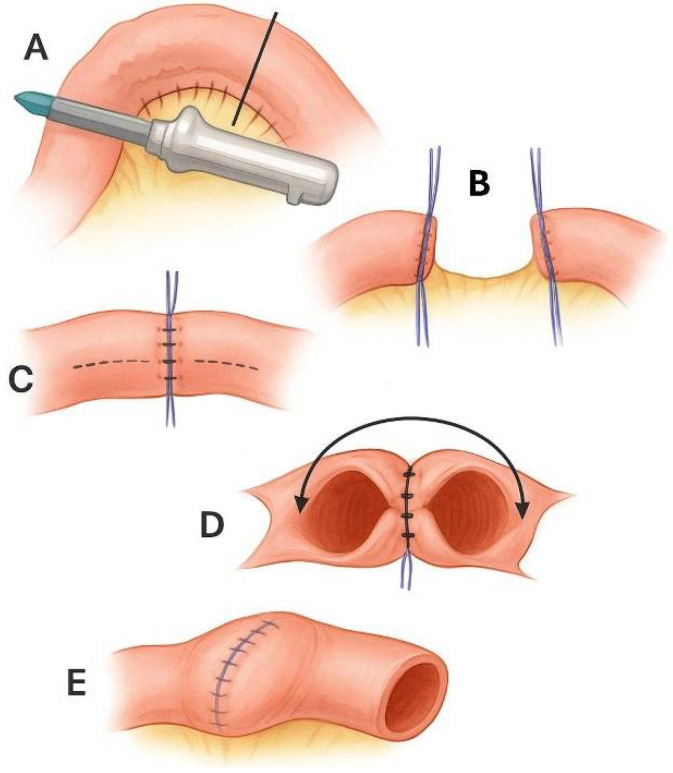
The Kono-S anastomosis procedure. (**A**) Bowel resection is performed using a linear stapler, ensuring that the mesenteric side is positioned centrally at the end of the stump. (**B**) Reinforcing sutures are placed along the cut edges of each bowel stump. (**C**) The two stumps are approximated and sutured together to form a central supporting column, helping preserve the anastomotic diameter and shape. Longitudinal enterotomies are made at the antimesenteric borders of both segments, as indicated by the dashed lines. (**D**) A side-to-side antimesenteric anastomosis is then constructed in the transverse orientation. (**E**) Upon completion, the supporting column lies between the mesentery and the anastomosis. This figure was created in part using DALL·E 3 (OpenAI).

Kono-S anastomosis was developed in response to the observation that recurrence often occurs on the mesenteric side of the anastomosis. It is a hand-sewn, antimesenteric, functional end-to-end anastomosis (Figure 3) [88]. Initial data from Kono’s group suggested reductions in both endoscopic and surgical recurrence, though this study was non-randomized and lacked a control arm.

Subsequent studies, including prospective and retrospective studies and one single-center randomized trial, have been published and meta-analyzed [89]. The randomized trial [90] reported a significant decrease in endoscopic recurrence using Kono-S anastomosis, although with a limited follow-up (6 months), a small sample size (79 patients), and other limitations. The meta-analysis yielded similar results, indicating a potential benefit with low complication rates. However, a more recent multicenter retrospective study by the GETAID surgical group [91], involving 433 patients and applying propensity score matching, found no significant difference in the recurrence rates between Kono-S and conventional anastomoses. In light of these conflicting results, two randomized controlled trials are ongoing [92]. An interim analysis from one of them, led by Trencheva et al. [93], including the first 250 patients, showed no difference in endoscopic recurrence at 3–6 months. This large, multicenter trial suggests that Kono-S anastomosis does not offer a clear advantage over the standard techniques.

Regarding extended mesenteric resection, retrospective data suggest that it may reduce postoperative recurrence. Two recent meta-analyses [94,95], including the same five studies (four retrospective and one randomized trial), reported reduced recurrence with extended resection. In contrast, a third meta-analysis showed the opposite results, indicating that there was no difference between extended and limited resection [96]. This is because the four favorable retrospective studies had much larger sample sizes than that in the SPICY study [97], which was the only randomized clinical trial. Its smaller sample size (139 patients) gives it a small statistical weight compared to that of the other studies. Despite this, its results, in which no differences are observed between the two techniques, should be given special consideration given the robust methodology.

In conclusion, although early retrospective data were promising, well-designed prospective randomized trials indicate that neither Kono-S anastomosis nor extended mesenteric resection significantly reduces postoperative recurrence in CD and therefore do not support changes in the current surgical practice.

## 7. Clinical Implications: Therapeutic Options Related to Obesity and Metabolic Syndrome in IBD

As described for obesity, the prevalence of metabolic syndrome and related conditions, such as type 2 diabetes, dyslipidemia, and hypertension, is rising worldwide, including among individuals with IBD. Similar to obesity, these comorbidities are associated with low-grade systemic inflammation. Moreover, several pharmacological treatments commonly used to manage these conditions have shown anti-inflammatory properties in vitro, in animal models, and in other inflammatory diseases, suggesting potential relevance in the context of IBD [98,99].

Research on the interaction between metabolic comorbidities and IBD has primarily focused on two areas: first, the association between specific treatments for metabolic conditions and the risk of developing IBD, and second, the impact of these treatments on the clinical course of established IBD. Most available studies share a similar design, relying on large population-based databases and retrospective analyses. As a result, they are subject to multiple sources of bias. Additionally, these studies typically assess isolated variables without accounting for confounding factors such as concomitant medications, obesity, smoking, or lifestyle.

Statins, which inhibit HMG-CoA reductase, have demonstrated potential anti-inflammatory effects. In vitro models of UC have shown that statins can modulate the TNF-α expression in intestinal epithelial cells [100,101]. Statins display anti-inflammatory and immunomodulatory effects on the intestinal mucosa beyond their lipid-lowering properties. By inhibiting the mevalonate pathway, they reduce the activation of NF-κB and the production of pro-inflammatory cytokines, while downregulating Th1/Th17 responses and enhancing regulatory T-cell activity. Statins also strengthen the epithelial tight junctions, limit oxidative stress, and may favorably influence gut microbiota composition [101,102,103]. Three prominent studies have evaluated their role in IBD. Lochhead et al. [104] and Ungaro et al. [105] reported a significantly reduced risk of IBD among statin users. In contrast, Khalil et al. [106] did not find a significant association. All three studies used retrospective population-based data and focused on a single exposure variable, limiting their interpretability.

Metformin, a biguanide widely used for type 2 diabetes mellitus, has also been investigated. Beyond glycemic control, it exhibits pleiotropic anti-inflammatory effects and contributes to maintaining intestinal barrier integrity, as demonstrated in various experimental models. In these studies, it has been found that metformin exerts notable modulatory effects on the gut microbiota and intestinal barrier function. Evidence from both animal and human studies indicates that metformin promotes the expansion of beneficial bacterial taxa, including *Akkermansia muciniphila* and *Bifidobacterium* spp., which are associated with increased production of short-chain fatty acids and anti-inflammatory activity. In parallel, metformin enhances epithelial tight junction integrity, thereby reducing intestinal permeability and the translocation of lipopolysaccharides [107,108,109,110]. Retrospective studies in human cohorts have reported inconsistent findings. Tseng [111] and Petrov et al. [112] suggested a reduced risk of IBD in metformin users with type 2 diabetes, whereas Allin et al. [113], focusing on older adults, did not observe a significant association. As with statins, these studies are limited by their retrospective design and narrow focus on isolated variables.

GLP-1 receptor agonists have gained particular attention recently [114]. Preclinical studies suggest that these agents may reduce systemic inflammation through both indirect mechanisms, such as weight loss, and direct effects, including modulation of intraepithelial lymphocyte activity via GLP-1 receptor signaling in the gut [115]. Mouse models have shown the upregulation of IL-33, mucin 5b, and CCL20 expression, which are decreased in IBD [116]. In addition, it has also been suggested that GLP-1 agonists may increase the expression of genes linked to the tight junctions, improving intestinal barrier status and decreasing bacterial translocation [117]. A meta-analysis of randomized trials across different disease settings found that GLP-1 agonists lowered circulating TNF-α and CRP levels [118]. Several observational studies in humans have explored the impact of GLP-1 receptor agonists in IBD. Villumsen et al. [119] reported a reduced risk of flares or complications. Desai et al. [120] found no differences in disease outcomes, except for fewer hospitalizations among treated patients. Gorelik et al. [121] observed an improved disease course, while Nielsen et al. [122] evaluated only the ileus or obstruction risk and found no group differences. Despite their methodological heterogeneity and differences in outcomes, most studies report some clinical benefit without evidence of harm. Other studies, including those by St-Pierre et al. [123], Levine et al. [124], Anderson et al. [125], and Belinchón et al. [126], were limited by small sample sizes and insufficient power to draw firm conclusions. Overall, the clinical evidence remains limited and heterogeneous, especially in terms of the outcomes measured by each study. Furthermore, potential confounding factors such as concurrent therapies, the baseline disease activity, and obesity-related comorbidities complicate the interpretation of these findings. The most consistent finding to date is that GLP-1 agonists are not associated with worsened IBD. Currently, randomized controlled trials are underway to assess the combined efficacy of tirzepatide and IL-23 inhibitors in patients with IBD, both CD and UC (NCT06937099).

In conclusion, while the preliminary findings are encouraging, especially regarding GLP-1 receptor agonists and metformin, higher-quality prospective studies are needed. These should comprehensively assess the interplay between metabolic syndrome, its pharmacological management, and IBD outcomes.

## 8. Conclusions

Chronic inflammation mediated by obesity and the intestinal inflammation characteristic of IBD converge in common pathophysiological mechanisms, such as intestinal dysbiosis, persistent immune activation, and pro-inflammatory cytokine production, along with intestinal barrier dysfunction. The current evidence suggests that obesity may be a risk factor for developing CD and possibly aggravating the clinical course of IBD, although conflicting findings exist due to methodological complexities.

The adipose tissue, especially mesenteric fat or “creeping fat”, plays an active role in IBD (particularly in CD), serving both as an indicator of severe transmural disease and as a participant in the local inflammatory response through mediator secretion and interaction with the affected intestine. However, attempts to act therapeutically, especially from the surgical perspective, have not met the promising expectations they initially generated.

Another potential action pathway lies in using medications targeting metabolic pathologies, such as statins, metformin, and GLP-1 analogs, which could act by modulating inflammation. Preclinical and observational data support their possible beneficial effects on IBD onset and progression, but the existing studies are limited by retrospective designs, confounding factors, and heterogeneous outcomes.

In light of these limitations, there is a need for prospective, high-quality studies that address the complex interactions between obesity, metabolic syndrome, and IBD. These studies should consider the role of adipose tissue dysfunction, the gut microbiota, lifestyle factors, and the potential of metabolic drugs as adjunctive therapies in IBD management.

## Figures and Tables

**Table 1:** A summary of the main epidemiological studies on the relationship between obesity and the development of IBD.

Author	Year	Study Design	Data Source	N	Obesity Measurement	Patient Characteristics	Outcome for Inflammatory Bowel Disease
Cañete [58]	2025	Population-based retrospective cohort	Catalan Health Surveillance System (CHSS)	1,117,427	Clinical diagnosis of obesity/severe obesity and bariatric surgery	General adult population	Increased risk of severe obesity with CD and UC
Chan [56]	2013	Prospective cohort	European Prospective Investigation into Cancer and Nutrition (EPIC)	300,724	BMI measured at baseline	General adult population	No association
Harpsoe [57]	2014	Prospective cohort	Danish National Birth Cohort (DNBC)	75,008	Self-reported pre-pregnancy BMI	General adult population	No association
Jensen [55]	2018	Prospective cohort	Copenhagen School Health Records Register (CSHRR)	316,799	BMI z-score between ages 7 and 13	Pediatric population	Inverse association with UC and direct association with CD
Khalili [53]	2015	Prospective cohort	Nurses’ Health Study II (EE.UU.)	111,498	Current BMI, BMI at age 18, weight, body shape, and waist and hip measurements	Nurses	Increased risk of CD
Mendall [54]	2018	Prospective cohort	Danish National Birth Cohort (DNBC)	74,512	Pre-pregnancy BMI and BMI 18 months postpartum	Pregnant women	Increased risk of CD

BMI: body mass index; N: sample size; CD: Crohn’s disease; UC: ulcerative colitis.

## Data Availability

Not applicable.

## Abbreviations

The following abbreviations are used in this manuscript:

IBD	Inflammatory Bowel Disease
CD	Crohn’s Disease
UC	Ulcerative Colitis
SCFAs	Short-Chain Fatty Acids
TNF	Tumor Necrosis Factor
IL	Interleukin
BMI	Body Mass Index
